# Denosumab treatment is associated with the absence of circulating tumor cells in patients with breast cancer

**DOI:** 10.1186/s13058-018-1067-y

**Published:** 2018-11-20

**Authors:** Marcus Vetter, Julia Landin, Barbara Maria Szczerba, Francesc Castro-Giner, Sofia Gkountela, Cinzia Donato, Ilona Krol, Ramona Scherrer, Catharina Balmelli, Alexandra Malinovska, Alfred Zippelius, Christian Kurzeder, Viola Heinzelmann-Schwarz, Walter Paul Weber, Christoph Rochlitz, Nicola Aceto

**Affiliations:** 1 Gynecologic Cancer Center, University Hospital Basel, 4056 Basel, Switzerland; 2 Department of Medical Oncology, University Hospital Basel, 4056 Basel, Switzerland; 3 Department of Biomedicine, Cancer Metastasis Laboratory, University of Basel and University Hospital Basel, Mattenstrasse 28, CH-4058 Basel, Switzerland; 4 SIB Swiss Institute of Bioinformatics, 1015 Lausanne, Switzerland; 5 Breast Center, University Hospital Basel, 4056 Basel, Switzerland

**Keywords:** Circulating tumor cells, Circulating tumor cell clusters, Denosumab, Breast cancer, Metastasis

## Abstract

**Background:**

The presence of circulating tumor cells (CTCs) in patients with breast cancer correlates to a bad prognosis. Yet, CTCs are detectable in only a minority of patients with progressive breast cancer, and factors that influence the abundance of CTCs remain elusive.

**Methods:**

We conducted CTC isolation and enumeration in a selected group of 73 consecutive patients characterized by progressive invasive breast cancer, high tumor load and treatment discontinuation at the time of CTC isolation. CTCs were quantified with the Parsortix microfluidic device. Clinicopathological variables, blood counts at the time of CTC isolation and detailed treatment history prior to blood sampling were evaluated for each patient.

**Results:**

Among 73 patients, we detected at least one CTC per 7.5 ml of blood in 34 (46%). Of these, 22 (65%) had single CTCs only, whereas 12 (35%) featured both single CTCs and CTC clusters. Treatment with the monoclonal antibody denosumab correlated with the absence of CTCs, both when considering all patients and when considering only those with bone metastasis. We also found that low red blood cell count was associated with the presence of CTCs, whereas high CA 15-3 tumor marker, high mean corpuscular volume, high white blood cell count and high mean platelet volume associated specifically with CTC clusters.

**Conclusions:**

In addition to blood count correlatives to single and clustered CTCs, we found that denosumab treatment associates with most patients lacking CTCs from their peripheral circulation. Prospective studies will be needed to validate the involvement of denosumab in the prevention of CTC generation.

**Electronic supplementary material:**

The online version of this article (10.1186/s13058-018-1067-y) contains supplementary material, which is available to authorized users.

## Background

Circulating tumor cells (CTCs) are derivatives of solid tumor lesions that detach from the tumor and enter the bloodstream [1]. In patients with breast cancer, CTCs have been shown to be predictive of a shorter disease-free survival and overall survival [2, 3], with a worse prognosis in patients who present with a count of at least five CTCs per 7.5 ml of blood [2, 3]. Generally, high CTC counts have been associated with a poor prognosis in multiple settings, including those patients that are newly diagnosed with metastatic breast cancer and about to start a therapy [3, 4]. At the morphological level, breast CTCs occur in the blood of patients as single CTCs or as CTC clusters, with the latter being associated with a shorter metastasis-free survival than in patients in whom only single CTCs are found [5].

Although the association between CTCs and bad prognosis is well established in breast cancer, CTCs are detectable only in a subset (~ 20–40%) of patients [2, 5]. To date, no parameters have been found that could explain CTC abundance in patients, leading to difficulties in enabling patient stratification prior to CTC-related investigations [6, 7], as well as limiting our understanding of those factors that may influence the spread of cancer.

In this study, we aimed to investigate a number of clinicopathological variables, blood counts at the time of CTC isolation and detailed treatment history prior to blood sampling in a cohort of 73 consecutive patients with invasive breast cancer characterized by progressive disease, high tumor load and treatment discontinuation (or without any pretreatment) at the time of CTC isolation, before the next line of therapy. Additionally, we not only investigated parameters that are associated with the presence of CTCs but also specifically interrogated our datasets to identify features that are associated with CTC clusters. The rationale of our study was therefore to identify, in an unbiased manner (i.e., not driven by preexisting hypotheses), clinical parameters that correlate with CTC presence in patients with progressive breast cancer.

## Methods

### Patient selection

Seventy-three consecutive patients with invasive breast cancer, progressive disease, high tumor load, treatment discontinuation at the time of CTC isolation (before the next line of therapy) and no preselection for breast cancer subtype or specific metastatic sites were enrolled in the study. Eligible patients were > 18 years old with any menopausal status and had an Eastern Cooperative Oncology Group performance status of 0–3. Disease had to be measurable by Response Evaluation Criteria in Solid Tumors (RECIST) version 1.1 or nonmeasurable bone-only disease. Tumor load was defined by either the size of the primary tumor or the number and size of metastatic lymph nodes or distant sites, and patients with higher tumor load were prioritized. All subjects donated 7.5–15 ml of blood in ethylenediaminetetraacetic acid (EDTA) vacutainers at least once, and each signed an informed consent before joining the study. The study was performed under the protocols EKNZ BASEC 2016-00067 and EK 321/10, which received ethical and institutional review board approvals before study initiation (Ethics Committee northwest/central Switzerland [EKNZ]). This study was performed in compliance with the Declaration of Helsinki.

### CTC isolation and enumeration strategy

Patient-derived CTCs were captured on the microfluidic Parsortix Cell Separation Cassette (GEN3D6.5; ANGLE, Guildford, UK) within 1 h of blood draw, directly from unmanipulated blood samples. Next, in-cassette staining was performed with an antibody cocktail comprising antibodies against epithelial cell adhesion molecule (EpCAM)-Alexa Fluor 488 (AF488) (#CST5198; Cell Signaling Technology, Danvers, MA, USA), human epidermal growth factor receptor 2 (HER2)-AF488 (#324410; BioLegend, San Diego, CA, USA), epidermal growth factor receptor (EGFR)-fluorescein isothiocyanate (FITC) (#GTX11400; GeneTex, Irvine, CA, USA) and CD45-BV605 (#304042; BioLegend). CTCs were characterized as AF488/FITC-positive and BV605-negative and enumerated manually by two independent operators under a fluorescence microscope at 20× magnification.

### Clinical parameter assessment

Primary tumor samples were collected at the initial diagnosis, and IHC was performed for estrogen receptor (ER), progesterone receptor (PR), HER2 and Ki-67. If the patient had primary metastatic disease, a biopsy from the metastatic site was obtained when possible, including marker assessment: ER, PR and HER2. Histopathological diagnosis was conducted by two independent pathologists from the breast cancer unit at the University Hospital Basel. All patients were treated at the Breast Cancer Unit University Hospital Basel according to local standard operating procedures and National Comprehensive Cancer Network and European Society for Medical Oncology guidelines by senior breast oncologists. If a patient had a progression within new distant sites, a new biopsy from that site was taken, when possible, to determine ER, PR and HER2. Patients under systemic treatment had tumor assessment at least every 12 weeks with computed tomographic scans or earlier if tumor progression was anticipated. CTC collection was performed at progression and prior to the next line of therapy or before any treatment was conducted. The patients’ data was retrieved by detailed retrospective chart review. Data collection included demographics and disease-specific and treatment-specific data including age, gender, primary stage, histologic subtype, ER/PR/HER2 status, grading, Ki-67, date of primary diagnosis and relapse, type of relapse (localized, metastatic), site of distant disease, bone-modifying agents (bisphosphonates, denosumab), palliative irradiation, and type of systemic treatment, including time on treatment and time to next subsequent treatment. Data was correlated with CTC counts.

### Blood parameter assessment

Complete blood counts were measured with the ADVIA 120 Hematology Analyzer (Siemens Healthcare Diagnostics, Tarrytown, NY, USA) using Multispecies version 5.9.0-MS software (Bayer Diagnostics, Tarrytown, NY, USA). Blood samples were taken before each new therapy cycle or at least every month, including cancer antigen 15-3 (CA 15-3), alkaline phosphatase, Ca^2+^, C-reactive protein, lactate dehydrogenase, red blood cells (RBC), hemoglobin, hematocrit, mean corpuscular volume (MCV), mean corpuscular hemoglobin concentration, white blood cells (WBC), neutrophils, lymphocytes, monocytes, eosinophils, basophils, large unstained cells, platelets and mean platelet volume (MPV). In the vast majority of cases, blood samples were taken simultaneously with the CTC sample or within 7 days after CTCs were taken. Eight of 73 patients had only partial data available, whereas no blood counts were reported at the time of CTC detection for nine of 73 patients.

### Statistical analysis

We first screened our data to exclude variables and patients with high content of missing information, as well as observations with implausible values. Cancer therapies were simplified into three main nonexclusive categories (targeted therapy, chemotherapy and hormone therapy) (Additional file 1: Table S1). Some patients had undergone multiple lines of therapy. For this reason, we assessed the effects of accumulated therapies and the therapy at CTC evaluation separately.

We investigated the association between the different variables of interest and the presence of CTCs using Fisher’s exact test for categorical variables, two-sided Wilcoxon rank-sum test for continuous variables (e.g., complete blood counts) and Kruskal-Wallis test for ordinal variables with more than two levels (e.g., stage at diagnosis). A list of the statistical tests used for each variable can be found in Additional file 2: Table S2. For each test, we present the nominal *P* value. An estimate and 95% CI are also provided for continuous and two-level categorical variables. The estimate corresponds to the OR in Fisher’s exact test and to the estimated median of the difference between samples from both groups in the Wilcoxon rank-sum test. To account for potential confounding variables, logistic regression analysis was conducted, adjusting by age at primary diagnosis, tumor stage at diagnosis, tumor grade and histologic subtype. Adjusted *P* values were calculated following the Benjamini-Hochberg method, combining all tests performed in this work. Associations with an adjusted *P* value ≤ 0.05 are highlighted in the text. We conducted the data wrangling and statistical analysis in R (version 3.4.0; R Foundation for Statistical Computing, Vienna, Austria).

## Results

### Patient characteristics

Given previously reported correlations between number of CTCs and tumor load [8], as well as the findings that CTC counts predict poor prognosis in breast cancer [3, 4, 9], we focused on a group of 73 consecutive patients with invasive breast cancer with the following characteristics: high tumor load, detailed treatment history available, progressive disease associated with treatment discontinuation at the time of CTC isolation (before the next line of therapy), and availability of comprehensive blood counts performed at CTC collection. Selected patients ranged from 36 to 85 years of age and carried either invasive ductal, lobular or inflammatory carcinoma, with a broad expression range of ER, PR, HER2 and Ki-67 protein levels, as well as tumor grade varying from 1 to 3. Detailed characteristics of patients, therapies and statistical tests used for the analysis are listed in Additional file 1: Table S1, Additional file 2: Table S2, and Additional file 3: Table S3.

### CTC isolation and enumeration

Blood samples were drawn in EDTA vacutainers and processed directly with the Parsortix microfluidic device [10], with a dedicated protocol enabling the isolation of > 99% of breast CTCs from unlabeled blood samples (Additional file 4: Figure S1A). Upon enrichment, CTCs were stained with an antibody cocktail against EpCAM, EGFR and HER2 and counterstained for the WBC marker CD45 (Additional file 4: Figure S1A and B). With this approach, we detected at least one CTC per 7.5 ml of peripheral blood in 34 (46.6%) patients. Among these, we observed that 22 (64.7%) patients were characterized by the presence of single CTCs and that 12 (35.3%) patients had both single CTCs and CTC clusters (Additional file 4: Figure S1C).

### Features of patients with CTCs

We investigated a number of clinicopathological variables to identify features associated with patients in whom either single CTCs or CTC clusters were found, compared with patients with no detectable CTCs. We first observed that previous treatments with targeted therapy (including but not limited to hormonal, anti-HER2, anti-CDK4/6 treatments), chemotherapy or radiotherapy did not correlate with the presence of CTCs (Additional file 5: Table S4). Yet, we found that treatment with the anti-bone resorption antibody denosumab (received by 21 of 73 patients) was associated with the absence of CTCs (OR, 0.25; 95% CI, 0.06–0.86; *P* = 0.019). Namely, the prevalence of CTCs was 14.7% (5 of 21) among patients treated with denosumab and 55.8% (29 of 52) among nontreated patients (Table 1). Further, when considering only those patients in whom CTCs were detected, the average CTC number was 9.8 for patients treated with denosumab (*n* = 5) versus 24.79 for nontreated patients (*n* = 29). Despite their role as anti-bone resorption agents, the same association was not seen for bisphosphonates (*P* = 0.784). Importantly, anti-bone resorption treatment with either denosumab or bisphosphonates was decided on the basis of treatment initiation date (denosumab was approved in Switzerland in December 2011 and given as the preferred treatment option to eligible patients after that date), whereas patients who started receiving bisphosphonates (i.e., prior to December 2011) continued receiving bisphosphonates unless major side effects occurred (Additional file 6: Table S5). When we restricted the analysis to the 44 patients with bone metastasis, denosumab was administered to 20 of 44 of them, and it also correlated with a reduction in CTC numbers compared with the remaining 24 patients with bone metastasis but no denosumab treatment (OR, 0.22; 95% CI, 0.04–0.96; *P* = 0.03) (Table 2). These results were confirmed using logistic regression adjusting by age at primary diagnosis, tumor stage at diagnosis, tumor grade and histologic subtype (OR, 0.25; 95% CI, 0.06–0.82; *P* = 0.03). When comparing clinicopathological variables in patients who were treated or not with denosumab, as expected, we observed a correlation with bone metastasis (OR, 22.53; 95% CI, 3.14–995.64; *P* = 5.6e-05; adjusted *P* = 0.01) (Additional file 7: Table S6), but no effect on progression-free survival was seen (Additional file 8: Figure S2). Together, our data show that denosumab treatment is associated with a marked reduction of CTC counts in patients with breast cancer.

**Table 1:** Clinical features of patients with circulating tumor cells

	No CTC (*n* = 39)	CTC (*n* = 34)	*P* value	Estimate (95% CI)
Age at primary diagnosis, years, mean (SD)	58.38 (11.85)	55.1 (11.04)	0.444	− 2.76 (− 8.49, 2.84)
Age at first CTC evaluation, years, mean (SD)	63.53 (11.69)	59.58 (10.7)	0.163	− 4.11 (− 9.27, 1.63)
Stage at diagnosis, *n* (%)			0.679	–
I	4 (10.53%)	5 (14.71%)		
IA	1 (2.63%)	0 (0%)		
II	5 (13.16%)	4 (11.76%)		
IIA	1 (2.63%)	4 (11.76%)		
III	11 (28.95%)	7 (20.59%)		
IIIA	2 (5.26%)	0 (0%)		
IIIC	0 (0%)	2 (5.88%)		
IV	14 (36.84%)	11 (32.35%)		
Lymphocyte node involvement, *n* (%)			0.881	–
N0	11 (31.43%)	8 (25.81%)		
N1	11 (31.43%)	14 (45.16%)		
N2	6 (17.14%)	0 (0%)		
N3	6 (17.14%)	9 (29.03%)		
Histologic subtype, *n* (%)			0.964	–
Invasive lobular	6 (15.38%)	4 (11.76%)		
Invasive ductal	31 (79.49%)	29 (85.29%)		
Inflammatory invasive lobular	1 (2.56%)	1 (2.94%)		
Inflammatory	2 (5.13%)	2 (5.88%)		
% of ER^+^ cells, mean (SD)	65.82 (42.48)	59.79 (40.93)	0.386	0 (− 10, 0)
% of PR^+^ cells, mean (SD)	38.97 (40.04)	26.35 (33.78)	0.171	− 4 (− 20, 0)
% of Ki-67^+^ cells, mean (SD)	27.08 (17.13)	31.59 (21.55)	0.514	5 (− 5, 10)
HER2^+^	7 (17.95%)	7 (22.58%)	0.766	1.33 (0.35–5.11)
Triple-negative	4 (10.81%)	3 (9.68%)	1.000	0.89 (0.12–5.73)
Tumor grade			0.985	–
1	4 (10.53%)	4 (12.12%)		
2	17 (44.74%)	14 (42.42%)		
3	17 (44.74%)	15 (45.45%)		
Bisphosphonates	9 (23.68%)	7 (20.59%)	0.784	0.84 (0.23–2.94)
Denosumab	16 (41.03%)	5 (14.71%)	0.019	0.25 (0.06–0.86)
Radiotherapy	22 (56.41%)	13 (38.24%)	0.160	0.48 (0.17–1.35)
Relapse				
Any	31 (79.49%)	25 (73.53%)	0.589	0.72 (0.21–2.45)
Local	3 (7.69%)	4 (11.76%)	0.698	1.59 (0.25–11.72)
Metastasis	26 (66.67%)	19 (55.88%)	0.470	0.64 (0.22–1.82)
Days between primary diagnosis and relapse, mean (SD)	1954.08 (2042.25)	1893.48 (1853.86)	0.966	− 9.37 (− 1000, 756)
Established metastatic disease at CTC evaluation	35 (89.74%)	30 (88.24%)	1.000	0.86 (0.15–5.04)
Number of metastatic sites, mean (SD)	2.09 (1.01)	1.9 (0.94)	0.473	0 (− 1, 0)
Metastasis site, *n* (%)				
Bone	27 (69.23%)	17 (51.52%)	0.150	0.45 (0.15–1.28)
Liver	10 (25.64%)	12 (36.36%)	0.447	1.57 (0.51–4.9)
Lymph node	9 (23.08%)	10 (30.3%)	0.599	1.38 (0.43–4.54)
Pleural	7 (17.95%)	2 (6.06%)	0.162	0.29 (0.03–1.68)
Peritoneal	3 (7.69%)	4 (12.12%)	0.698	1.59 (0.25–11.72)
Lung	4 (10.26%)	4 (12.12%)	1.000	1.16 (0.2–6.83)
Skin	3 (7.69%)	0 (0%)	0.243	0 (0–2.74)
Brain	2 (5.13%)	2 (6.06%)	1.000	1.15 (0.08–16.76)
Uterus	1 (2.56%)	1 (3.03%)	1.000	1.15 (0.01–92.67)
Muscular	1 (2.56%)	2 (6.06%)	0.595	2.35 (0.12–143.61)

*Abbreviations: ER* Estrogen receptor, *HER2* Human epidermal growth factor receptor 2, *PR* Progesterone receptor

The table shows clinical features of patients with and without circulating tumor cells (CTCs)

**Table 2:** Circulating tumor cells detection according to denosumab treatment and bone metastasis

	Number of samples	No CTCs	CTCs	*P* value	Estimate (95% CI)	*P* value	Estimate (95% CI)
Reference	28	12 (43%)	16 (57%)	Reference		–	–
Bone metastasis	24	11 (46%)	13 (54%)	1.000	0.89 (0.26–3.05)	Reference	
Bone metastasis and denosumab	20	16 (80%)	4 (20%)	0.017	0.19 (0.04–0.81)	0.030	0.22 (0.04–0.96)

The table shows the number of patients with and without circulating tumor cells (CTCs) among individuals with bone metastasis who were treated or not with denosumab

### Features of patients with CTC clusters

We further asked whether any clinicopathological variables might be associated specifically with the presence of CTC clusters, compared with patients in whom CTC clusters were not found (i.e., having either single CTCs or no CTCs). We found that both younger age at primary diagnosis and younger age at first CTC evaluation were associated with the presence of CTC clusters (Table 3). Particularly, we observed average ages at primary diagnosis of 50.63 years (SD, 12.60) for patients with CTC clusters and 58.08 years (SD, 10.99) for patients with no CTC clusters (*P* = 0.033), as well as average ages at first CTC evaluation of 54.87 years (SD, 12.14) for patients with CTC clusters and 63.03 years (SD, 10.77) for patients with no CTC clusters (*P* = 0.025). We also observed that although HER2 was expressed in 22% (13 of 61) of patients with no CTC clusters (7 of 39) (i.e., 17.95% of patients with no CTCs and 6 of 22 [30%] of patients with single CTCs only), it was expressed in only 1 patient (9.09%) with CTC clusters (OR, 0.36; 95% CI, 0.01–2.97; *P* = 0.44), even though the relationship between HER2 negativity and CTC clusters did not reach statistical significance (Table 3).

**Table 3:** Clinical features of patients with circulating tumor cell clusters

	No CTC clusters (*n* = 61)	CTC clusters (*n* = 12)	*P* value	Estimate (95% CI)
Age at primary diagnosis, years, mean (SD)	58.08 (10.99)	50.63 (12.6)	0.033	− 8.26 (− 15.3, − 0.44)
Age at first CTC evaluation, years, mean (SD)	63.03 (10.77)	54.87 (12.14)	0.025	− 8.3 (− 16.06, − 1.04)
Stage at diagnosis, *n* (%)			0.726	–
I	7 (11.67%)	2 (16.67%)		
IA	1 (1.67%)	0 (0%)		
II	7 (11.67%)	2 (16.67%)		
IIA	4 (6.67%)	1 (8.33%)		
III	16 (26.67%)	2 (16.67%)		
IIIA	2 (3.33%)	0 (0%)		
IIIC	1 (1.67%)	1 (8.33%)		
IV	21 (35%)	4 (33.33%)		
Lymphocyte node involvement, *n* (%)			0.855	–
N0	15 (27.27%)	4 (36.36%)		
N1	22 (40%)	3 (27.27%)		
N2	6 (10.91%)	0 (0%)		
N3	11 (20%)	4 (36.36%)		
Histologic subtype, *n* (%)			0.679	–
Invasive lobular	9 (14.75%)	1 (8.33%)		
Invasive ductal	49 (80.33%)	11 (91.67%)		
Inflammatory invasive lobular	1 (1.64%)	1 (8.33%)		
Inflammatory	3 (4.92%)	1 (8.33%)		
% of ER^+^ cells, mean (SD)	62.34 (41.8)	66.42 (42.1)	0.675	0 (− 10, 20)
% of PR^+^ cells, mean (SD)	32.71 (37.79)	34 (37.3)	0.888	0 (− 10, 20)
% of Ki-67^+^ cells, mean (SD)	30 (19.65)	23 (16.43)	0.384	− 5 (− 20, − 10)
HER2^+^, *n* (%)	13 (22.03%)	1 (9.09%)	0.442	0.36 (0.01–2.97)
Triple-negative, *n* (%)	7 (12.28%)	0 (0%)	0.588	0 (0–3.7)
Tumor grade, *n* (%)			0.093	–
1	5 (8.33%)	3 (27.27%)		
2	26 (43.33%)	5 (45.45%)		
3	29 (48.33%)	3 (27.27%)		
Bisphosphonates, *n* (%)	14 (23.33%)	2 (16.67%)	1.000	0.66 (0.06–3.68)
Denosumab, *n* (%)	19 (31.15%)	2 (16.67%)	0.489	0.45 (0.04–2.41)
Radiotherapy, *n* (%)	30 (49.18%)	5 (41.67%)	0.756	0.74 (0.17–3.06)
Relapse, *n* (%)				
Any	47 (77.05%)	9 (75%)	1.000	0.9 (0.19–5.83)
Local	4 (6.56%)	3 (25%)	0.082	4.61 (0.58–32.62)
Metastasis	40 (65.57%)	5 (41.67%)	0.193	0.38 (0.08–1.59)
Days between primary diagnosis and relapse, mean (SD)	1969.49 (2003.96)	1636.67 (1538.48)	0.633	− 236.86 (− 1643, 1203)
Established metastatic disease at CTC evaluation, *n* (%)	54 (88.52%)	11 (91.67%)	1.000	1.42 (0.15–70.01)
Number of metastatic sites, mean (SD)	1.96 (0.98)	2.18 (0.98)	0.452	0 (0–1)
Metastasis site, *n* (%)				
Bone	37 (61.67%)	7 (58.33%)	1.000	0.91 (0.22–4.08)
Liver	19 (31.67%)	3 (25%)	1.000	0.74 (0.12–3.42)
Lymph node	15 (25%)	4 (33.33%)	0.497	1.52 (0.29–6.74)
Pleural	9 (15%)	0 (0%)	0.339	0 (0–2.57)
Peritoneal	5 (8.33%)	2 (16.67%)	0.323	2.21 (0.19–16.05)
Lung	7 (11.67%)	1 (8.33%)	1.000	0.7 (0.01–6.47)
Skin	3 (5%)	0 (0%)	1.000	0 (0–12.81)
Brain	3 (5%)	1 (8.33%)	0.521	1.74 (0.03–24.14)
Uterus	1 (1.67%)	1 (8.33%)	0.304	5.27 (0.06–433.34)
Muscular	2 (3.33%)	1 (8.33%)	0.421	2.63 (0.04–54.78)

*Abbreviations: ER* Estrogen receptor, *HER2* Human epidermal growth factor receptor 2, *PR* Progesterone receptor

The table shows clinical features of patients with and without circulating tumor cell clusters (CTC clusters)

When considering only patients with CTCs and comparing those with CTC clusters versus those with single CTCs, we found that also in this context younger age at primary diagnosis (*P* = 0.044) and younger age at first CTC evaluation (*P* = 0.058) were associated with the presence of CTC clusters (Additional file 9: Table S7).

### Blood parameters associated with CTCs

In addition to investigating the clinical parameters summarized above, for each patient, we also evaluated comprehensive blood counts performed at CTC collection. We first asked whether blood-related parameters were associated with the presence of CTCs (either single or clustered), compared with patients in whom CTCs were not detected. We observed that patients with detectable CTCs had a lower RBC count (OR, − 0.42; 95% CI, − 0.8 - -0.08; *P* = 0.019) than patients with no CTCs (Table 4).

**Table 4:** Complete blood counts in patients with circulating tumor cells

	No CTC (*n* = 39)	CTC (*n* = 34)	*P* value	Estimate (95% CI)
CA 15-3, mean (SD)	223.71 (384.68)	1084.15 (4136.87)	0.658	6.7 (− 19.2, 87.6)
Alkaline phosphatase, mean (SD)	105.47 (103.98)	198.15 (365.58)	0.401	6 (− 12, 27)
Calcium (korr), mean (SD)	2.34 (0.15)	2.32 (0.25)	0.145	− 0.06 (− 0.13, 0.02)
CRP, mean (SD)	31.92 (47.56)	26.87 (47.69)	0.982	0 (− 8.8, 3.8)
LDH, mean (SD)	281.61 (118.18)	300.15 (228.57)	0.772	− 5 (− 36, 23)
RBC, 10^12^/L, mean (SD)	4.37 (0.56)	3.85 (0.77)	0.019	− 0.42 (− 0.8, − 0.08)
HGB, g/L, mean (SD)	130.14 (19.85)	118.15 (24.11)	0.051	− 11 (− 21, 0)
HCT, L/L, mean (SD)	0.38 (0.06)	0.35 (0.06)	0.053	− 0.03 (− 0.06, 0)
MCV, fl, mean (SD)	87.46 (5.68)	89.73 (5.26)	0.227	2 (− 1, 4)
MCH, pg, mean (SD)	29.62 (2.52)	30.66 (1.96)	0.157	0.8 (− 0.3, 1.8)
MCHC, g/L, mean (SD)	339.03 (13.98)	341.19 (12.79)	0.667	2 (− 6, 8)
WBC, 10^9^/L, mean (SD)	7.35 (2.13)	7.24 (3.53)	0.334	− 0.6 (− 1.88, 0.81)
Neutrophils, 10^9^/L, mean (SD)	5.33 (1.87)	5.12 (2.87)	0.239	− 0.63 (− 1.65, 0.56)
Lymphocytes, 10^9^/L, mean (SD)	1.37 (0.67)	1.41 (0.85)	0.941	− 0.02 (− 0.38, 0.38)
Monocytes, 10^9^/L, mean (SD)	0.42 (0.12)	0.44 (0.2)	0.843	− 0.01 (− 0.08, 0.07)
Eosinophils, 10^9^/L, mean (SD)	0.16 (0.18)	0.17 (0.12)	0.423	0.02 (− 0.03, 0.09)
Basophils, 10^9^/L, mean (SD)	0.04 (0.07)	0.04 (0.03)	0.256	0.01 (− 0.01, 0.02)
LUC, 10^9^/L, mean (SD)	0.15 (0.22)	0.12 (0.08)	0.912	0 (− 0.03, 0.02)
PLT, 10^9^/L, mean (SD)	289.92 (139.86)	249.93 (91.52)	0.277	− 30 (− 84, 24)
MPV, fl, mean (SD)	8.2 (1.51)	8.68 (1.52)	0.109	0 (0–1)

*Abbreviations: CA 15-3* Cancer antigen 15-3, *CRP* C-reactive protein, *HCT* Hematocrit, *HGB* Hemoglobin, *LDH* Lactate dehydrogenase, *LUC* Large unstained cells, *MCH* Mean corpuscular hemoglobin, *MCHC* Mean corpuscular hemoglobin concentration, *MCV* Mean corpuscular volume, *MPV* Mean platelet volume, *PLT* Platelets, *RBC* Red blood cells, *WBC* White blood cells

The table shows complete blood counts in patients with and without circulating tumor cells (CTCs).

### Blood parameters associated with CTC clusters

We then asked whether specific blood-related parameters could be associated with the presence of CTC clusters, compared with patients with no CTC clusters (i.e., having either no CTCs or single CTCs only). In this case, we found that patients with CTC clusters have 14-fold higher levels of the CA 15-3 tumor marker (*P* = 0.021), higher MCV (*P* = 0.033), higher WBC (*P* = 0.03) and higher MPV (*P* = 0.032) than patients in whom CTC clusters were not found (Table 5). We also restricted this analysis to patients with CTCs and compared patients with CTC clusters with patients with only single CTCs. In this setting, we further confirmed that patients with CTC clusters have 38-fold higher CA 15-3 tumor antigen (*P* = 0.0089), as well as nearly twofold higher total WBC counts (*P* = 0.0045) and higher neutrophil counts (*P* = 0.03) (Additional file 10: Table S8).

**Table 5:** Complete blood counts in patients with circulating tumor cell clusters

	No CTC clusters (*n* = 61)	CTC clusters (*n* = 12)	*P* value	Estimate (95% CI)
CA 15-3, mean (SD)	172.5 (324.45)	2554.6 (6387.64)	0.021	204.16 (9.7–515)
Alkaline phosphatase, mean (SD)	106.4 (104.27)	310.25 (525.57)	0.301	10 (− 12, 74.58)
Calcium (korr), mean (SD)	2.33 (0.14)	2.36 (0.35)	0.698	− 0.02 (− 0.16, 0.14)
CRP, mean (SD)	27.76 (44.47)	37.25 (58.6)	0.279	2.9 (− 4, 21.4)
LDH, mean (SD)	271 (102.59)	363.33 (329.24)	0.463	17 (− 26, 76)
RBC, 10^12^/L, mean (SD)	4.26 (0.6)	3.66 (0.93)	0.078	− 0.51 (− 1.16, 0.06)
HGB, g/L, mean (SD)	127.52 (20.18)	114.18 (29.19)	0.183	− 12 (− 30, 6)
HCT, L/L, mean (SD)	0.38 (0.05)	0.34 (0.08)	0.159	− 0.03 (− 0.09, 0.01)
MCV, fl, mean (SD)	87.7 (5.58)	91.73 (4.41)	0.033	4 (0–7)
MCH, pg, mean (SD)	29.8 (2.42)	31.24 (1.51)	0.064	1.1 (0–2.3)
MCHC, g/L, mean (SD)	339.8 (13.38)	340.64 (14.25)	0.646	2 (− 10, 10)
WBC, 10^9^/L, mean (SD)	6.87 (2.25)	9.38 (3.99)	0.030	2.54 (0.26–4.68)
Neutrophils, 10^9^/L, mean (SD)	4.94 (1.87)	6.65 (3.6)	0.177	1.22 (− 0.52, 3.68)
Lymphocytes, 10^9^/L, mean (SD)	1.38 (0.69)	1.42 (1)	0.756	− 0.11 (− 0.62, 0.6)
Monocytes, 10^9^/L, mean (SD)	0.41 (0.13)	0.5 (0.24)	0.336	0.06 (− 0.08, 0.26)
Eosinophils, 10^9^/L, mean (SD)	0.16 (0.16)	0.17 (0.14)	0.974	0 (− 0.07, 0.1)
Basophils, 10^9^/L, mean (SD)	0.04 (0.06)	0.04 (0.04)	0.983	0 (− 0.02, 0.02)
LUC, 10^9^/L, mean (SD)	0.14 (0.19)	0.12 (0.11)	0.471	− 0.01 (− 0.05, 0.02)
PLT, 10^9^/L, mean (SD)	278.02 (125.8)	251.5 (109.99)	0.667	− 15.84 (− 94, 47)
MPV, fl, mean (SD)	8.32 (1.62)	8.82 (0.87)	0.032	1 (0–1)

*Abbreviations: CA 15-3* Cancer antigen 15-3, *CRP* C-reactive protein, *HCT* Hematocrit, *HGB* Hemoglobin, *LDH* Lactate dehydrogenase, *LUC* Large unstained cells, *MCH* Mean corpuscular hemoglobin, *MCHC* Mean corpuscular hemoglobin concentration, *MCV* Mean corpuscular volume, *MPV* Mean platelet volume, *PLT* Platelets, *RBC* Red blood cells, *WBC* White blood cells

The table shows complete blood counts in patients with and without circulating tumor cell clusters (CTC clusters)

## Discussion

In a selected cohort of 73 patients with progressive invasive breast cancer, we provide a detailed description of a number of clinicopathological parameters and blood counts at the time of CTC isolation that correlate with the presence of single CTCs and CTC clusters. Interestingly, we observed that treatment with the monoclonal antibody denosumab in patients with bone metastasis strongly correlated with the absence of CTCs from their peripheral circulation, suggesting a scenario in which the treatment itself might influence CTC spread from the bone tissue. Importantly, this correlation is not seen regarding treatment with the anti-bone resorption drug bisphosphonate, possibly because of different administration routes or dosing schedules [11] or, alternatively, potential off-target binding of denosumab to proteins other than receptor activator of nuclear factor κB ligand (RANKL).

Although its focus was on clinical parameters, our study did not provide molecular insights into the mechanism of action of denosumab in the context of its role in inhibiting CTC generation. Yet, considering that most denosumab-treated patients are characterized by bone metastatic disease but no primary breast tumor (which has been surgically removed prior to denosumab treatment), CTCs represent derivatives of their bone metastatic lesions. In this setting, we speculate that the effect of denosumab in suppressing CTC generation could be a result of RANKL inhibition within the bone, preventing the maturation of preosteoclasts into osteoclasts [12] and protecting the bone from degradation, leading to a lower likelihood of a bone metastatic lesion to shed CTCs. However, we cannot exclude an action of denosumab on breast cancer cells themselves, which have previously been shown to express high receptor activator of nuclear factor κB (RANK) levels [13, 14] and may be susceptible to its inhibition. Prospective studies and molecular assays will be needed to specifically dissect the role and mechanism of action of denosumab in CTC generation.

Recently, a phase 3 clinical trial designed to determine the long-term effects of denosumab treatment (D-CARE; ClinicalTrials.gov, NCT01077154) showed no benefits in metastasis-free survival and overall survival of patients with breast cancer. Importantly, individuals within this study were mainly patients with early breast cancer (i.e., stage IIB to IIIC), while our patient cohort was largely dominated by patients with stage IV disease. Although we are not aware of CTC enumeration data being evaluated within the D-CARE study, it is possible that denosumab might play a different role in the intravasation of bone metastasis-derived CTCs (as seen in our study) as opposed to primary tumor-derived CTCs (D-CARE).

Among other correlations, we observed an intriguing association between the absence of HER2 expression in the primary tumor and the presence of CTC clusters. Although this result did not reach statistical significance, our observation regarding HER2 does not seem to be influenced by the metastatic tropism of HER2-positive breast cancers, and it might reveal important insights into the signaling networks involving CTC cluster formation, also considering HER2 expression fluctuations in CTCs and breast cancer metastasis [15, 16]. In other words, we speculate that HER2 signaling might influence cancer cells to intravasate as single CTCs, whereas its absence might point them toward collective invasion into the bloodstream. This hypothesis will require experimental testing.

We also found that CTC clusters, but not CTCs in general, are more prevalent in younger patients. Both CTC clusters and younger age have been associated with worse prognosis and reduced survival rates [2, 5, 17–20]. In this case, it is unlikely that younger age represents an independent risk factor for CTC cluster formation, but rather it may reflect an association with tumor aggressiveness [21].

Last, blood counts at the time of CTC collection provide evidence for applying well-established, cost-effective and widespread blood-testing strategies to stratify patients with higher likelihood to present with detectable CTCs. For instance, we find that lower RBC count has good correlation with the presence of CTCs. Additionally, CA 15-3 tumor antigen is highly increased in patients with CTC clusters, possibly reflecting a higher tumor load but also tumors that are characterized by an elevated shedding of mucin 1 (MUC-1)-containing cells into the bloodstream [22]. A functional relationship between MUC-1 and CTC clusters remains to be investigated. We also observed that higher MCV, higher MPV and higher WBC counts correlate with the presence of CTC clusters. We envision these parameters to be used to stratify patient populations to conduct CTC-related studies in the setting of advanced breast cancer.

Altogether, our study is meant as an exploratory analysis to evaluate the association of multiple clinical predictors with the presence of CTCs. Given the high number of hypotheses tested and the relatively low number of patient samples in the study (*n* = 73), none of the associations reported show a *P* value less than 0.05 after adjustment for multiple comparisons, with the exception of the correlation between denosumab treatment and the presence of bone metastasis (adjusted *P* = 0.01). For this reason, subsequent prospective and experimental studies should be conducted to validate the associations that are presented in this work, including the role of denosumab in CTC shedding.

## Conclusions

Our data provide evidence of the association between treatment with the monoclonal antibody denosumab and the absence of CTCs from the peripheral circulation of patients with breast cancer. This finding suggests that denosumab treatment may be beneficial to reduce cancer spread in patients who are diagnosed with bone metastasis.

Although factors such as limited blood volume and diverse CTC isolation technologies may influence CTC detection rate in patients with cancer, the identification of a set of clinical correlatives to CTCs in breast cancer is likely to facilitate the identification of those patients who would benefit the most from CTC analysis, including genetic profile assessment for patient stratification [6, 23] and testing of drug susceptibility [7]. As an added benefit to this analysis, the identification of denosumab treatment as a strategy to reduce CTC intravasation warrants further investigation.

## Additional files

Additional file 1: **Table S1.** Drug classification. The table shows the drug classification used for the analysis, grouping drugs into targeted therapy, chemotherapy, hormone therapy and immunotherapy. *Abbreviations: cbx6* Chromobox 6, *CDK4* Cyclin-dependent kinase 4, *CDK6* Cyclin-dependent kinase 6, *EGFR* Epidermal growth factor receptor, *Her2* Human epidermal growth factor receptor 2, *mTOR* Mechanistic target of rapamycin, *PD-L1* Programmed cell death 1 ligand 1, *VEGF* Vascular endothelial growth factor. (XLSX 10 kb)

Additional file 2: **Table S2.** Variable classification and statistical test applied. The table shows the type of variable and statistical test used for the analysis of individual clinicopathological parameters. *Abbreviations: ER*, Estrogen receptor, *HER2*, Human epidermal growth factor receptor 2, *PR* Progesterone receptor. (XLSX 10 kb)

Additional file 3: **Table S3.** Patient characteristics. The table shows the characteristics of the 73 patients included in the study. *Abbreviations: ER* Estrogen receptor, *HER2* Human epidermal growth factor receptor 2, *ID* Invasive ductal, *IL* Invasive lobular, *NA* Not available, *PR* Progesterone receptor, *ECOG* Eastern Cooperative Oncology Group (as defined by Oken et al. [24]). (XLSX 14 kb)

Additional file 4: **Figure S1.** Circulating tumor cell (CTC) capture strategy. (**a**) Schematic drawing showing the size-based capturing principle of the Parsortix microfluidic device (*left*). Plot showing the capture efficiency of the Parsortix microfluidic device for MCF7 cells spiked in healthy blood samples (*right*). Representative images of a captured MCF7 single cell, a cell cluster (*green*) and a contaminant white blood cell (WBC; red) in the Parsortix microfluidic cassette (*bottom*). (**b**) Representative images of a captured single CTC, a CTC cluster (*green*) and a contaminant WBC (*red*) from a breast cancer patient sample. (**c**) Bar graph showing the number of patients in whom no CTCs, single CTCs or CTC clusters were found. (PDF 245 kb)

Additional file 5: **Table S4.** Therapy evaluation in patients with circulating tumor cells. The table shows the types of therapy that patients with and without circulating tumor cells (CTCs) underwent. (XLSX 9 kb)

Additional file 6: **Table S5.** Bisphosphonates or denosumab treatment. The table shows whether bisphosphonates or denosumab was administered to each of the patients included in the study. (XLSX 10 kb)

Additional file 7: **Table S6.** Clinical features of patients who were treated or not with denosumab. The table shows clinical features of patients who were treated or not with denosumab. *Abbreviations: ER* Estrogen receptor, *HER2* Human epidermal growth factor receptor 2, *PR* Progesterone receptor. (XLSX 11 kb)

Additional file 8: **Figure S2.** Progression-free survival of patients who were treated or not with denosumab. Kaplan-Meier curve showing the progression-free survival probability of patients who were treated (*red*) or not (*green*) with denosumab (*top*). *P* = 0.95 by pairwise log-rank test. The table shows the number of patients at each time point (*bottom*). (PDF 188 kb)

Additional file 9: **Table S7.** Clinical features of patients with single circulating tumor cell and circulating tumor cell clusters. The table shows clinical features of patients in whom only single circulating tumor cells (CTC single cell) or also clustered circulating tumor cells (CTC clusters) were found. *Abbreviations: ER* Estrogen receptor, *HER2* Human epidermal growth factor receptor 2, *PR* Progesterone receptor. (XLSX 11 kb)

Additional file 10: **Table S8.** Complete blood counts in patients with single circulating tumor cells and circulating tumor cell clusters. The table shows complete blood counts in patients in whom only single circulating tumor cells (single CTC) or also clustered circulating tumor cells (CTC clusters) were found. *Abbreviations: CA 15-3* Cancer antigen 15-3, *CRP* C-reactive protein, *HCT* Hematocrit, *HGB* Hemoglobin, *LDH* Lactate dehydrogenase, *LUC* Large unstained cells, *MCH* Mean corpuscular hemoglobin, *MCHC* Mean corpuscular hemoglobin concentration, *MCV* Mean corpuscular volume, *MPV* Mean platelet volume, *PLT* Platelets, *RBC* Red blood cells, *WBC* White blood cells. (XLSX 10 kb)

## Abbreviations

AF488: Alexa Fluor 488
CA 15-3: Cancer antigen 15-3
CRP: C-reactive protein
CTC: Circulating tumor cell
EDTA: Ethylenediaminetetraacetic acid
EGFR: Epidermal growth factor receptor
EpCAM: Epithelial cell adhesion molecule
ER: Estrogen receptor
FITC: Fluorescein isothiocyanate
HCT: Hematocrit
HER2: Human epidermal growth factor receptor 2
HGB: Hemoglobin
LDH: Lactate dehydrogenase
LUC: Large unstained cells
MCH: Mean corpuscular hemoglobin
MCHC: Mean corpuscular hemoglobin concentration
MCV: Mean corpuscular volume
MPV: Mean platelet volume
MUC-1: Mucin 1
PLT: Platelets
PR: Progesterone receptor
RANK: Receptor activator of nuclear factor κB
RANKL: Receptor activator of nuclear factor κB ligand
RBC: Red blood cells
WBC: White blood cells

## References

[1] Aceto N, Toner M, Maheswaran S, Haber DA (2015). En route to metastasis: circulating tumor cell clusters and epithelial-to-mesenchymal transition. Trends Cancer.

[2] Rack B, Schindlbeck C, Jückstock J, Andergassen U, Hepp P, Zwingers T (2014). Circulating tumor cells predict survival in early average-to-high risk breast cancer patients. J Natl Cancer Inst.

[3] Cristofanilli M, Budd GT, Ellis MJ, Stopeck A, Matera J, Miller MC (2004). Circulating tumor cells, disease progression, and survival in metastatic breast cancer. N Engl J Med.

[4] Giuliano M, Giordano A, Jackson S, Hess KR, De Giorgi U, Mego M (2011). Circulating tumor cells as prognostic and predictive markers in metastatic breast cancer patients receiving first-line systemic treatment. Breast Cancer Res.

[5] Aceto N, Bardia A, Miyamoto DT, Donaldson MC, Wittner BS, Spencer JA (2014). Circulating tumor cell clusters are oligoclonal precursors of breast cancer metastasis. Cell.

[6] Carter L, Rothwell DG, Mesquita B, Smowton C, Leong HS, Fernandez-Gutierrez F (2017). Molecular analysis of circulating tumor cells identifies distinct copy-number profiles in patients with chemosensitive and chemorefractory small-cell lung cancer. Nat Med.

[7] Yu M, Bardia A, Aceto N, Bersani F, Madden MW, Donaldson MC (2014). Cancer therapy: ex vivo culture of circulating breast tumor cells for individualized testing of drug susceptibility. Science.

[8] Kaifi JT, Kunkel M, Dicker DT, Joude J, Allen JE, Das A (2015). Circulating tumor cell levels are elevated in colorectal cancer patients with high tumor burden in the liver. Cancer Biol Ther.

[9] Cristofanilli M, Broglio KR, Guarneri V, Jackson S, Fritsche HA, Islam R (2007). Circulating tumor cells in metastatic breast cancer: biologic staging beyond tumor burden. Clin Breast Cancer.

[10] Xu L, Mao X, Imrali A, Syed F, Mutsvangwa K, Berney D (2015). Optimization and evaluation of a novel size based circulating tumor cell isolation system. PLoS One.

[11] Whitaker M, Guo J, Kehoe T, Benson G (2012). Bisphosphonates for osteoporosis—where do we go from here?. N Engl J Med.

[12] Hanley DA, Adachi JD, Bell A, Brown V (2012). Denosumab: mechanism of action and clinical outcomes. Int J Clin Pract.

[13] Blake ML, Tometsko M, Miller R, Jones JC, Dougall WC (2014). RANK expression on breast cancer cells promotes skeletal metastasis. Clin Exp Metastasis.

[14] Pfitzner BM, Branstetter D, Loibl S, Denkert C, Lederer B, Schmitt WD (2014). RANK expression as a prognostic and predictive marker in breast cancer. Breast Cancer Res Treat.

[15] Jordan NV, Bardia A, Wittner BS, Benes C, Ligorio M, Zheng Y (2016). HER2 expression identifies dynamic functional states within circulating breast cancer cells. Nature.

[16] Houssami N, Macaskill P, Balleine RL, Bilous M, Pegram MD (2011). HER2 discordance between primary breast cancer and its paired metastasis: tumor biology or test artefact? Insights through meta-analysis. Breast Cancer Res Treat.

[17] Anderson WF, Pfeiffer RM, Dores GM, Sherman ME (2006). Comparison of age distribution patterns for different histopathologic types of breast carcinoma. Cancer Epidemiol Biomark Prev.

[18] Adami HO, Malker B, Holmberg L, Persson I, Stone B (1986). The relation between survival and age at diagnosis in breast cancer. N Engl J Med.

[19] Fredholm H, Magnusson K, Lindstrom LS, Garmo H, Falt SE, Lindman H (2016). Long-term outcome in young women with breast cancer: a population-based study. Breast Cancer Res Treat.

[20] Bleyer A, Barr R, Hayes-Lattin B, Thomas D, Ellis C, Anderson B (2008). The distinctive biology of cancer in adolescents and young adults. Nat Rev Cancer.

[21] Koleckova M, Kolar Z, Ehrmann J, Korinkova G, Trojanec R (2017). Age-associated prognostic and predictive biomarkers in patients with breast cancer. Oncol Lett.

[22] Duffy MJCA (1999). 15-3 and related mucins as circulating markers in breast cancer. Ann Clin Biochem.

[23] Lohr JG, Adalsteinsson VA, Cibulskis K, Choudhury AD, Rosenberg M, Cruz-Gordillo P (2014). Whole-exome sequencing of circulating tumor cells provides a window into metastatic prostate cancer. Nat Biotechnol.

[24] Oken MM, Creech RH, Tormey DC, Horton J, Davis TE, McFadden ET, Carbone PP (1982). Toxicity and response criteria of the Eastern Cooperative Oncology Group. Am J Clin Oncol..

