# Supplementary material for: Denosumab treatment is associated with the absence of circulating tumor cells in patients with breast cancer
Source: Breast Cancer Res. 2018 Nov 20;20:141. doi: 10.1186/s13058-018-1067-y (PMC6247738; doi:10.1186/s13058-018-1067-y)
Supplement: Supplementary file 4 — Figure S1. Circulating tumor cell (CTC) capture strategy. (a) Schematic drawing showing the size-based capturing principle of the Parsortix microfluidic device (left). Plot showing the capture efficiency of the Parsortix microfluidic device for MCF7 cells spiked in healthy blood samples (right). Representative images of a captured MCF7 single cell, a cell cluster (green) and a contaminant white blood cell (WBC; red) in the Parsortix microfluidic cassette (bottom). (b) Representative images of a captured single CTC, a CTC cluster (green) and a contaminant WBC (red) from a breast cancer patient sample. (c) Bar graph showing the number of patients in whom no CTCs, single CTCs or CTC clusters were found. (PDF 245 kb) [file 13058_2018_1067_MOESM4_ESM.pdf]

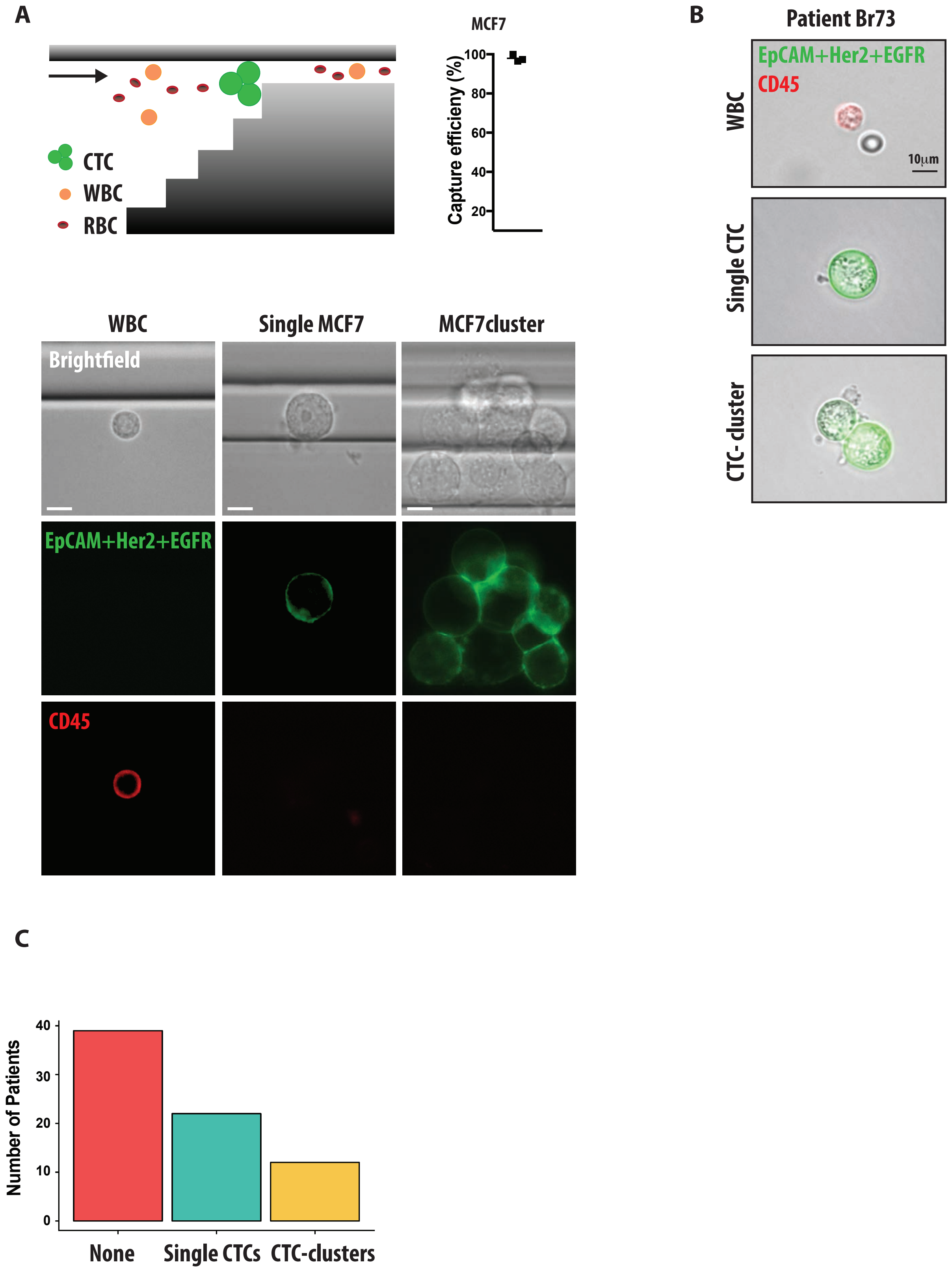

Supplementary Figure 1: Circulating tumor cell (CTC) capture strategy. (**A**) Schematic drawing showing the size-based capturing principle of the Parsortix microfluidic device (*left*). Plot showing the capture efficiency of the Parsortix microfluidic device for MCF7 cells spiked in healthy blood samples (*right*). Representative images of a captured MCF7 single cell, a cell cluster (green) and a contaminant white blood cell (WBC; red) in the Parsortix microfluidic cassette (*bottom*).
(**B**) Representative images of a captured single CTC, a CTC cluster (green) and a contaminant WBC (red) from a breast cancer patient sample. (**C**) Bar graph showing the number of patients in whom no CTCs, single CTCs or CTC clusters were found.
