# Supplementary material for: Denosumab treatment is associated with the absence of circulating tumor cells in patients with breast cancer
Source: Breast Cancer Res. 2018 Nov 20;20:141. doi: 10.1186/s13058-018-1067-y (PMC6247738; doi:10.1186/s13058-018-1067-y)
Supplement: Supplementary file 8 — Figure S2. Progression-free survival of patients who were treated or not with denosumab. Kaplan-Meier curve showing the progression-free survival probability of patients who were treated (red) or not (green) with denosumab (top). P = 0.95 by pairwise log-rank test. The table shows the number of patients at each time point (bottom). (PDF 188 kb) [file 13058_2018_1067_MOESM8_ESM.pdf]

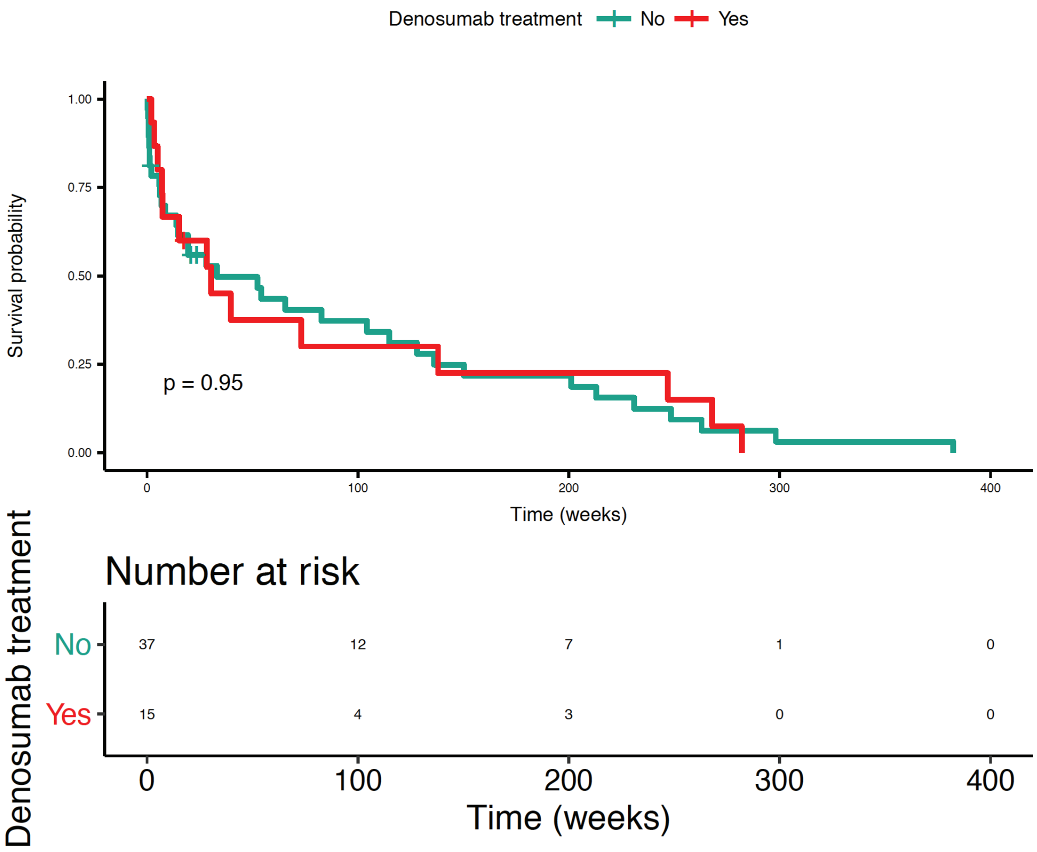

Supplementary Figure 2: Progression-free survival of patients that were treated or not with Denosumab. Kaplan-Meier curve showing the progression-free survival probability of patients that were treated (*red*) or not (*green*) with Denosumab (*top*). P=0.95 by paiwise Log-Rank test. The table shows the number of patients at each timepoint (*bottom*).
